# Genome-wide DNA methylation changes in skeletal muscle between young and middle-aged pigs

**DOI:** 10.1186/1471-2164-15-653

**Published:** 2014-08-05

**Authors:** Long Jin, Zhi Jiang, Yudong Xia, Ping’er Lou, Lei Chen, Hongmei Wang, Lu Bai, Yanmei Xie, Yihui Liu, Wei Li, Bangsheng Zhong, Junfang Shen, An’an Jiang, Li Zhu, Jinyong Wang, Xuewei Li, Mingzhou Li

**Affiliations:** Institute of Animal Genetics and Breeding, College of Animal Science and Technology, Sichuan Agricultural University, Ya’an, Sichuan 625014 China; Novogene Bioinformatics Institute, Beijing, 100083 China; E-GENE, Shenzhen, Guangdong 518173 China; Chongqing Academy of Animal Science, Chongqing, 402460 China; BGI-Tech, BGI-Shenzhen, Shenzhen, 518083 China

**Keywords:** DNA methylation, Skeletal muscle, Pig, Aging, MeDIP-seq, *DNMT*s

## Abstract

**Background:**

Age-related physiological, biochemical and functional changes in mammalian skeletal muscle have been shown to begin at the mid-point of the lifespan. However, the underlying changes in DNA methylation that occur during this turning point of the muscle aging process have not been clarified. To explore age-related genomic methylation changes in skeletal muscle, we employed young (0.5 years old) and middle-aged (7 years old) pigs as models to survey genome-wide DNA methylation in the *longissimus dorsi* muscle using a methylated DNA immunoprecipitation sequencing approach.

**Results:**

We observed a tendency toward a global loss of DNA methylation in the gene-body region of the skeletal muscle of the middle-aged pigs compared with the young group. We determined the genome-wide gene expression pattern in the *longissimus dorsi* muscle using microarray analysis and performed a correlation analysis using DMR (differentially methylated region)-mRNA pairs, and we found a significant negative correlation between the changes in methylation levels within gene bodies and gene expression. Furthermore, we identified numerous genes that show age-related methylation changes that are potentially involved in the aging process. The methylation status of these genes was confirmed using bisulfite sequencing PCR. The genes that exhibited a hypomethylated gene body in middle-aged pigs were over-represented in various proteolysis and protein catabolic processes, suggesting an important role for these genes in age-related muscle atrophy. In addition, genes associated with tumorigenesis exhibited aged-related differences in methylation and expression levels, suggesting an increased risk of disease associated with increased age.

**Conclusions:**

This study provides a comprehensive analysis of genome-wide DNA methylation patterns in aging pig skeletal muscle. Our findings will serve as a valuable resource in aging studies, promoting the pig as a model organism for human aging research and accelerating the development of comparative animal models in aging research.

**Electronic supplementary material:**

The online version of this article (doi:10.1186/1471-2164-15-653) contains supplementary material, which is available to authorized users.

## Background

Aging is a nearly universal, chronic process that is shared by all organisms. The most prominent feature of the aging process in mammals is a gradual loss of function at the cellular, tissue and organismal levels. ‘Aging epigenetics’ is an emerging field that has generated exciting revelations. A global loss of DNA methylation has been identified as an age-related epigenetic alteration [1]. Many studies have revealed that DNA methylation plays an important role in aging and in the development of various diseases [2, 3]. Therefore, a survey of epigenetic signatures that change with age might be useful to identify biomarkers of aging and age-associated diseases, which could potentially be used to make clinical diagnoses and prolong the lifespan.

The aging process and its underlying mechanisms have been studied extensively in rodent models [4, 5]. The sequencing and analysis of the pig genome [6] will greatly accelerate the development of the pig as a biomedical model for many diseases in humans, such as obesity and diabetes [7–9]. However, few aging studies have been carried out using pigs as models. Pigs age at a rate of approximately 5 years to every 1 year of human life, resulting in an average life expectancy of 15–20 years. Therefore, pigs could serve as an appealing model for studying aging because of their relatively longer lifespan than rodents and similar metabolic features, cardiovascular systems and proportional organ sizes relative to those of humans [10–12].

Notably, an increase in the incidence of age-related pathologies mostly begins at approximately the mid-point of a species’ life span [13–15]. It is well documented that the remarkable structural and functional changes that occur in skeletal muscle during aging, including a reduction of muscle mass and increased apoptosis [14, 16–19], are initiated at the mid-point of the lifespan [14]. Consequently, studies focusing on DNA methylation changes in skeletal muscle during the mid-life period compared with the young stage are long overdue.

In this study, we used the pig as a model to perform a genome-wide survey of differences in DNA methylation and gene expression in a representative skeletal muscle (*longissimus dorsi* muscle, LDM) between two age stages in female pigs: young (0.5 years old) and middle-aged (MA, 7 years old) [10, 11]. We identified the patterns of methylation in the pig genome and the age-related differentially methylated regions (DMRs), then performed functional enrichment analysis for genes exhibiting DMRs. We found more genes showing a hypomethylated gene body in the middle-aged pigs than in the young pigs; these genes were potentially involved in aging processes, such as the development of muscular atrophy. We believe that this study will serve as a valuable resource for aging studies while also promoting the pig as a model organism for human aging research and accelerating the considerable development of comparative animal models for aging research.

## Results

### Global DNA methylation analysis

**Figure 1:**
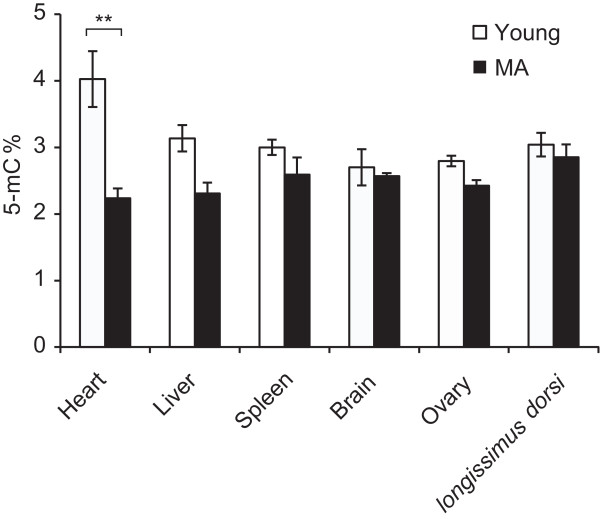
**Global methylation status of six tissues in young and middle-aged pigs.** “5-mC%” denotes the ratio of methylated cytosines to all nucleotides in the genome. (Student’s *t*-test, **P < 0.01).

Epigenetic alterations, such as global DNA hypomethylation, have been shown to progressively accumulate during aging [1]. We therefore first investigated the global DNA methylation status of six types of tissue by performing direct colorimetric quantification of methylated DNA. The global methylation level in the heart was significantly decreased in the MA pigs compared with the younger pigs (Figure 1), whereas the methylation levels in five other tissues showed a slight (but not significant) decrease in the MA pigs. This global loss of DNA methylation in older pigs was consistent with studies in humans, supporting a potential role of this type of epigenetic alteration in age-related gene regulation.

DNA methyltransferases (*DNMT*s) are crucial for the establishment and maintenance of genomic methylation patterns [20]. To determine whether the global loss of DNA methylation observed in the MA pigs was associated with alterations in *DNMTs*, we evaluated the mRNA transcript levels of *DNMT1* (the major maintenance methyltransferase), *DNMT3a* and *3b* (two *de novo* methylation methyltransferase) in these six tissues. For *DNMT1* and *DNMT3a*, no significant differences in gene expression were observed in most of the tissues between the two age groups (Additional file 1), whereas *DNMT3b* showed increased mRNA levels in most of the tissues (except the ovary) in the MA pigs. Previous studies have revealed increased expression of both *DNMT3a* and *DNMT3b* in aging fibroblasts and in the aging human liver [21, 22]. However, the expression level of *DNMT3a* showed no difference between young and MA pigs. Our result suggests that *DNMT3b*, rather than *DNMT3a*, may regulate the genomic methylation pattern in a tissue-specific manner in pigs during aging.

### Summary of methylated DNA immunoprecipitation sequencing (MeDIP-seq) data

Approximately 46 gigabases (Gb) of MeDIP-seq data were generated from six skeletal muscle samples (approximately 7.64 Gb per sample), among which 81% (approximately 35.31 Gb) of the clean reads were aligned to the porcine reference genome build 9.2. After removing ambiguous reads from the clean reads, 75% of the reads (approximately 36.56 Gb) were uniquely aligned across all of the aligned reads. The reads that showed the same mapping locations in each sample were regarded as potentially duplicated clones generated via PCR amplification and treated as the same read. Consequently, we acquired 24.46 Gb (approximately 92% of the unique mappable reads) of uniquely aligned non-duplicated reads (Additional file 2). CpG sites covered by a read depth of more than 10 were scored as high-confidence CpG sites. On average, 34.32% of the CpG sites met this threshold (Additional file 3).

To study methylation changes on a genome-wide scale, we measured methylation levels along the chromosomes in the samples using a 1 Mb sliding window to smooth the distribution (Additional file 4). Correlations between methylation levels and genomic features were assessed. We found that the methylation levels across the chromosomes were negatively correlated with the chromosomal length (Pearson’s *r =* 0.633, *P =* 0.0036) and positively correlated with the GC content (*r =* 0.787, *P =* 6.49 × 10^−5^), single-nucleotide polymorphism (SNP) density (*r =* 0.549, *P =* 0.0149) and gene density (*r* = 0.516, *P =* 0.0236). In addition, a strong positive correlation was observed with the ratio between the observed and expected numbers of CpG sites (CpG_o/e_) (*r* = 0.931, *P =* 7.41 × 10^−9^), which agreed with the results of a previous study of porcine DNA methylomes (Additional file 5) [8]. However, methylation levels were not correlated with the density of repeat regions (*r* = 0.348, *P =* 0.145). Among these genomic features, the CpG_o/e_ ratio showed the highest correlation with the methylation level. The gene density also exhibited a significant correlation with the methylation level, which may due to the relatively higher GC content in the gene regions being examined (Additional file 6), suggesting the potential role of methylation changes in the regulation of gene transcription [23]. Previous studies have demonstrated a strong genetic component of the variation in DNA methylation profiles [24], and a potential role has been suggested for CpG-SNPs in genetic variation of the epigenome [25]. Previous studies demonstrated that the level of methylation contributes to variation in the substitution rates at CpG sites [26, 27]. We observed a positive correlation between the SNP density and methylation level, suggesting that the genetic variation reflected by SNPs may have a substantial impact on local methylation patterns and gene expression.

We defined 24 categories of functional genomic elements and further classified the promoters into three types based on their CpG cites. Each type of promoter was then classified according to its distance from the transcription start site (Additional file 7) [8]. We also classified CpG islands and CGI shores into four categories according to their genomic locations, as described in previous studies [24, 28]. We found that intermediate CpG promoters (ICPs) exhibited a relatively higher methylation status than did high CpG promoters (HCPs) and low CpG promoters (LCPs) (two-way ANOVA, *P =* 2.87 × 10^−37^). The methylation levels within the distal (D), proximal (P) and intermediate (I) regions of promoters also showed significant differences (two-way ANOVA, *P =* 1.44 × 10^−7^) (Additional file 7). This result agreed with a previous finding that methylation occurs more frequently at ICPs [29]. Our data also suggested that a relatively higher methylation level within gene bodies is a general phenomenon in mammals [30], and it has been correlated with gene expression levels [28]. We observed that the methylation level of exons was higher than that of introns (*P* = 9.8 × 10^−9^) (Additional file 7), reflecting the higher GC content of exons compared with their surrounding introns and further indicating the possible different roles of exons and introns in the regulation of gene transcription [31]. Recently, DNA methylation at CpG island (CGI) shores has been demonstrated to play a more important role in gene regulation than that of the CGIs themselves [32]. We observed distinct methylation levels for CGI shores in various genomic locations (Additional file 7), which may suggest the distinctive roles of these CGI shores in regulating gene expression.

### Differential DNA methylation in the subtelomeric regions of young and MA pigs

**Figure 2:**
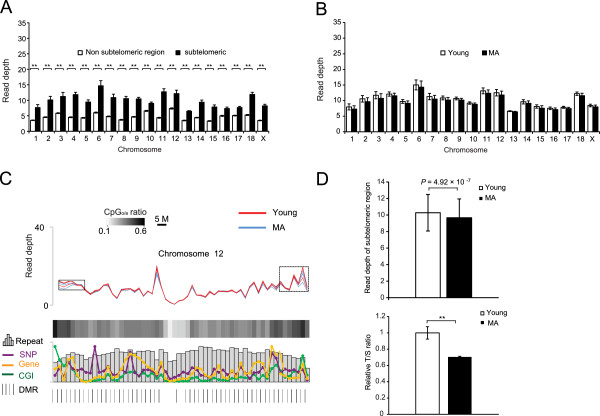
**Comparison of the methylation state between young and MA pigs across chromosomes.** Methylation levels in **(A)** subtelomeric versus non-subtelomeric regions and **(B)** the subtelomeric regions of young versus middle-aged pigs on each chromosome. Student’s *t*-test revealed no significant difference between the two groups. **(C)** Distribution of DNA methylation on pig chromosome 12. **(D)** Top: the average DNA methylation in subtelomeric regions in young versus middle-aged pigs. The significance was evaluated using a paired samples *t*-test (*P =* 4.92 × 10^−7^). Bottom: the T/S ratio (telomere signal versus the signal of the single-copy gene) reflects the relative telomere length in young and middle-aged pigs.

We surveyed the chromosomal profiles of DNA methylation and found that the subtelomeric regions showed significant hypermethylation compared with non-subtelomeric regions for all chromosomes (Figure 2A), which was consistent with the heavily methylated status of the subtelomeric region previously reported in mice [33]. Epigenetic modifications of the subtelomeric region correlate with telomere elongation, which is closely related to aging. Interestingly, we found that the methylation levels of the subtelomeric regions of each chromosome exhibited distinct patterns between the young and MA pigs (Figure 2B and 2C; Additional file 4). The average methylation status of the subtelomeric regions of all of the chromosomes of the MA pigs was significantly lower than that of the young pigs (Figure 2D). Previous studies of humans revealed that low levels of subtelomeric methylation may contribute toward increasing the levels of telomeric repeat-containing RNA, whose transcription originates in the subtelomere and is accompanied by a reduction in telomerase activity [34], thereby controlling the telomere length [35]. We therefore also measured telomere length using a qPCR assay (Figure 2D). The ratio of the telomere signal to the signal of the single-copy gene (T/S) (see “Methods”) was relatively lower in the MA pigs than in the young pigs, suggesting that the average telomere length was shorter in the MA pigs [36]. This result indicated that hypomethylation in the subtelomeric regions of the MA pigs may be accompanied by telomere attrition and involved in the aging process.

### Differentially methylated regions (DMRs) associated with aging

We identified 9,234 DMRs (with a Benjamini-Hochberg corrected *P* < 0.01, FDR < 0.01) in skeletal muscle between the young and MA pigs, representing approximately 0.064% of the length of the genome and approximately 0.28% of the total number of CpGs in the genome (Table 1). We performed hierarchical clustering of the samples using the DMRs of each genomic element and differentially expressed genes. Among all of the clustering plots, six samples could be clustered into two groups: young pigs and MA pigs, although the topological structure within the groups showed subtle differences (Additional file 8). This result revealed a high correlation between the biological replicates and indicated experimental reliability and relative epigenetic concordance within each group.

**Table 1:** **Summary of differentially methylated regions (DMRs)**

DMR type	Number of DMRs	Percentage of genomic length*	Percentage of genomic CpGs†
Age-related DMRs (*n* = 6)	9,234	0.064	0.280

*Total length of all DMRs relative to the length (approximately 2.60 billion bp) of the pig genome (Sscrofa 9.2).

†The number of CpGs among the total DMRs relative to the total number of CpGs (approximately 26.91 M) in the pig genome (Sscrofa 9.2).

**Figure 3:**
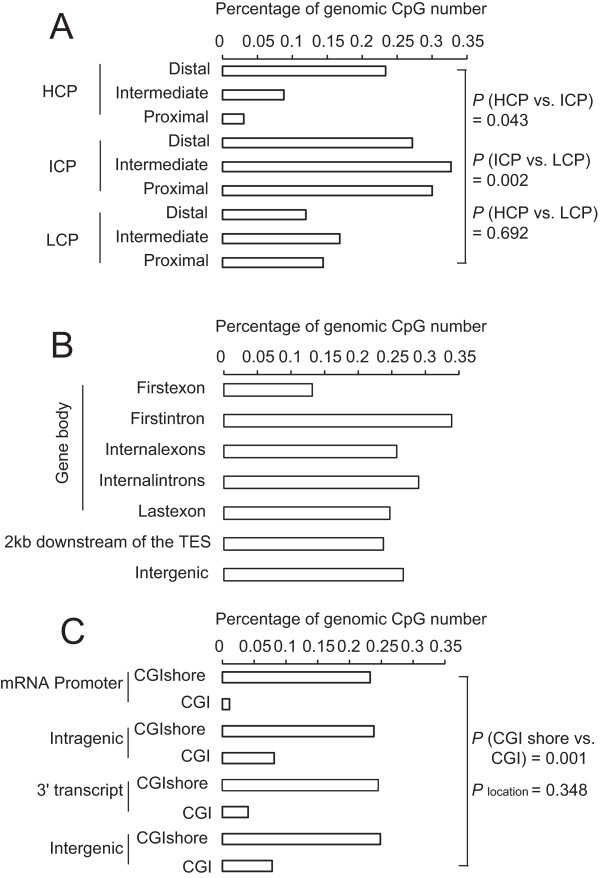
**Genome-wide distribution of differentially methylated regions.** The x-axis denotes the percentage of CpG sites within DMRs in each of 24 genomic elements (number of CpGs in DMRs versus the total number of CpGs in each of the genomic features). **(A)** Promoters were classified into nine categories based on their CpG representation (HCP, ICP and LCP) and genomic location (D, I, P). The significance of the pairwise comparisons among HCPs, ICPs and LCPs was evaluated using *t*-tests, whereas the CpG percentage showed no significant difference among the D, I and P promoters. **(B)** CpG percentages in various gene structures. **(C)** Comparison of CpG percentages between CGIs and CGI shores located in distinct genomic regions. The significance of the comparison of CpG percentages between CGIs and CGI shores was evaluated using two-way ANOVA.

To further explore potential distribution biases in the age-related DMRs, we analyzed the percentage of CpGs within the DMRs in each of the 24 genomic elements. Among promoters, more DMRs were enriched in the ICPs compared with HCPs and LCPs. ICPs generally contain weak CpG islands and are prone to regulation by DNA methylation [29]. Our results indicated that not only did ICPs exhibit the highest methylation levels compared with the other two promoter classes (Additional file 7), but DMRs were also observed more frequently in ICPs (Figure 3A). These observations demonstrated that ICPs are more susceptible to methylation and suggested that this type of alteration of the methylation status of ICPs may play an important role in modulating gene expression relevant to several biological processes, such as aging. Meanwhile, although a previous study found that the distal (D) regions of promoters contained more DMRs [8], no significant enrichment of DMRs was observed in the present study except in the distal regions of HCPs, suggesting that various types of DMRs may participate in distinct biological processes (Figure 3A). There is increasing evidence that methylation in the gene body affects gene expression in plants [37, 38] and mammals [30]. Our analysis indicated that more DMRs are located in gene bodies (1,720) than in promoters (185) (Additional file 9), which may suggest that gene bodies are more susceptible to changes compared to promoters during the aging process. The first exon contained relatively few DMRs within the gene body (Figure 3B), which may be the result of certain motifs overlapping between the promoter and the first exon. We also found that the majority of DMRs occurred at CGI shores (two-way ANOVA, *P =* 0.001), whereas the numbers of DMRs did not significantly vary across gene features (*P* = 0.348), consistent with previous reports on human cancer (Figure 3C) [32, 39–41].

### Gene-body DNA methylation and gene expression

**Figure 4:**
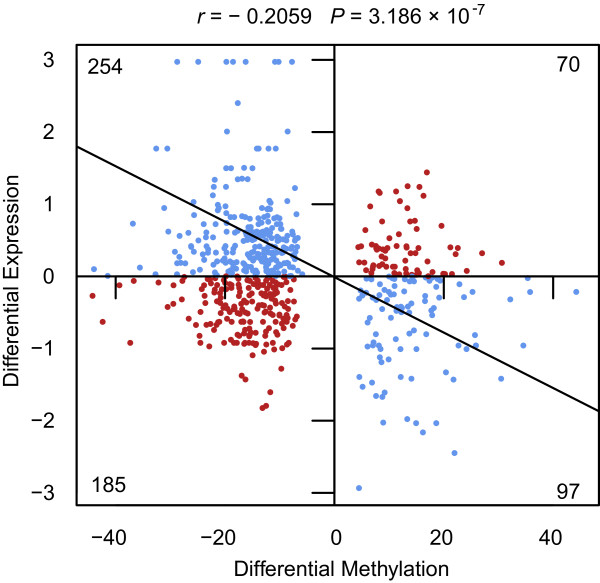
**Correlation of the methylation profile with expression levels.** Scatter plot and trend line (Pearson’s correlation) showing the correlation between the differences in mRNA expression and methylation. The line represents a linear regression. Blue and red dots represent the mRNA-differentially methylated region (DMR) pairs exhibiting either inverse or equivalent relationships, respectively.

The influence of the methylation status of promoter regions is considered to be an important mechanism regulating gene transcription. Nevertheless, investigation of the specific roles of DNA methylation in gene bodies is long overdue. To explore whether intragenic methylation affects gene expression, we performed a correlation analysis of DMR-mRNA pairs and observed a significant negative correlation (*r =* − 0.206, *P =* 3.186 × 10^−7^) between changes in the methylation levels in the gene body and gene expression (Figure 4). Whether methylation in the gene body inhibits or induces transcription remains unclear [30, 31, 37, 42]. Nevertheless, we conclude here that gene-body methylation reduced gene expression, possibly via an intragenic DNA methylation-induced decrease in the Pol II elongation efficiency [42].

### Functional enrichment analysis for genes with DMRs

**Figure 5:**
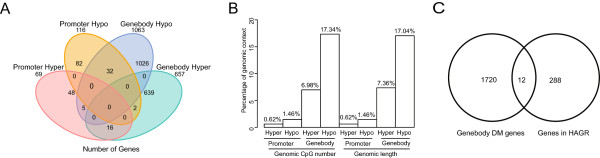
**Differentially methylated (DM) genes in gene bodies and promoters. (A)** Venn diagram of the numbers of genes showing promoter and gene-body DMRs. **(B)** Comparison of the genomic CpG percentage (number of CpGs in DMRs versus the total number of CpGs) and the genomic length percentage (DMR length versus the total length of DMRs) between gene-body and promoter regions. **(C)** Gene-body-DM genes that overlap with known age-related genes in the HAGR database.

To examine the potential function of the genes that showed a differential methylation status, we performed an enrichment analysis for genes with DMRs in their promoters and genes that were hyper- and hypomethylated in the gene body associated with age. Because DNA methylation concentrates at gene bodies on the X chromosome due to X chromosome inactivation (Xi), we excluded the DMRs on chromosome X from this analysis. A total of 185 genes that exhibited DMRs in their promoters were mapped to their human orthologs, and 657 and 1,063 genes with DMRs in their gene bodies that mapped to human genes showed hyper- and hypomethylation in the MA pigs, respectively (Figure 5A). More DMRs were observed in gene bodies rather than promoters, and the DMRs in gene bodies were more prone to be hypomethylated with age (Figure 5B). The finding that more genes were hypomethylated in the MA group also supported the theory of progressive global methylation loss during aging. The genes displaying DMRs in their promoters were not significantly enriched in any biological process or molecular function, which may due to the relatively low abundance of genes with DMRs in their promoters. The gene-body hypermethylated genes were significantly enriched for the processes ‘GTPase regulator activity’ (30 genes, *P* = 0.03), ‘ATP binding’ (79 genes, *P* = 0.03) and ‘protein kinase activity’ (39 genes, *P* = 0.04) (Additional file 10). Notably, the gene-body hypomethylated genes showed significant enrichment for various processes related to proteolysis (*P* = 4.55 × 10^−4^), protein catabolism (*P* = 5.84 × 10^−8^), and energy metabolism (GO: energy derivation by oxidation of organic compounds, *P* = 0.04) (Additional file 10). Previous studies have indicated that catabolic processes increase in aged muscles [43]. Our findings suggested that the hypomethylated genes observed in the *longissimus dorsi* muscle of the MA pigs were involved in protein degradation and may be responsible for muscular atrophy, which is one of the general structural and phenotypic changes observed in aging muscle [14].

### Genes involved in the aging process

To further highlight the potential roles of genes involved in aging, we considered the intersection of genes that presented DMRs in their promoters and gene bodies with the known age-related genes deposited in the Human Ageing Genomic Resources (HAGR) database [44]. Among the 288 genes potentially involved in the human aging process according to the HAGR-GenAge database, we did not identify any genes with DMRs in their promoters, whereas there were 12 known age-related genes included in the list of genes with DMRs in their gene bodies (Fisher’s exact test, *P =* 0.024) (Figure 5C).

**Figure 6:**
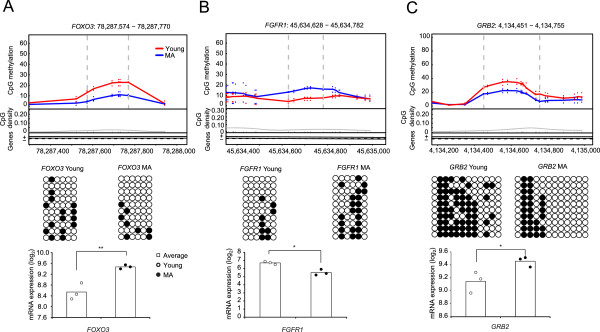
**Examples of age-related genes showing differential DNA methylation in their gene bodies.** **(A)** DMR in *FOXO3* gene body. **(B)** DMR in *FGFR1* gene body. **(C)** DMR in *GRB2* gene body. Top panels, Top half: CpG methylation. Each point represents methylation level (MeDIP-seq read depth) of a sample at a given CpG site. The curves showed average over the samples. The two vertical dashed lines marked the boundaries of the DMR identified. Lower half: CpG dinucleotides (black tick marks on X axis), CpG density (grey line). Plus and minus marks denote sense and antisense gene transcription. Second panels: validation of CpG methylation by bisulfite sequencing PCR (BSP). Ten subclones were selected for the BSP analysis. The solid circles represent the methylated CpG sites, and the open circles represent the unmethylated CpG sites. Third panels: comparison of gene expression levels between two age groups. The gene expression levels were taken from microarray data and normalized to the expression levels in young pigs.

A potential role of DNA methylation of the gene body in regulating gene expression has been proposed [28, 31, 37, 45]. We therefore investigated genes with DMRs in the gene body. Several genes included in the list of well-characterized human age-related genes exhibited differentially methylated gene bodies, which was confirmed using the bisulfite sequencing PCR (BSP) approach, and were shown to cause changes in mRNA expression (Figure 6). For example, *FoxO3* (forkhead box subfamily O member 3) has been well documented to be an essential transcription factor involved in the atrophy of muscles and myotubes [46]. *FoxO3* stimulates lysosomal proteolysis in muscle by activating autophagy and proteasomal pathways, and inducing the expression of autophagy-related genes [47]. We observed up-regulated expression and a lower gene-body methylation status of this gene in MA pigs (Figure 6A), in accordance with previous findings in aging muscle. Another gene, *FGFR1* (fibroblast growth factor receptor 1), which shows an opposite function to that of *FoxO3*, could inhibit the atrophy of skeletal muscle [48]. Down-regulated expression of *FGFR1*, together with hypermethylation of its gene body, was observed in MA pig skeletal muscle (Figure 6B). *GRB2* (growth factor receptor-bound protein 2) is critical for cell proliferation, and emerging evidence indicates that *GRB2* plays a role in tumorigenesis and is over-expressed in tumors [49]. In addition, a reduced expression level of *GRB2* in skeletal muscle contributes to increased insulin sensitivity [50, 51]. Microarray mRNA expression and genome-wide methylation data on *GRB2* revealed an increased expression level and hypomethylation in the gene body of this gene, respectively (Figure 6C).

## Discussion

This study provides a comprehensive analysis of genome-wide DNA methylation patterns in the skeletal muscle of aging pigs. Similar to previous reports in humans and mice, a global loss of methylation induced by transcriptional changes in *DNMT3b* was observed in various tissues of the MA pigs, suggesting that this type of epigenetic alteration is common in aging mammals. Although global DNA hypomethylation and promoter CpG island hypermethylation have been observed to progressively accumulate during aging [52], the present study identified more DMRs in gene bodies than in promoters, and gene-body hypomethylation was observed in more genes in the MA pigs (Figure 5 and Additional file 9). It is therefore reasonable to assume that during the loss of global methylation during aging, there is a greater tendency toward hypomethylation in the gene body rather than the promoter. It should be noted that based on our current data, it is not feasible to identify methylation changes on the X chromosome because of Xi [53]. Further research using SNP data could allow allele-specific analysis of DNA methylation to identify the specific methylation changes on chromosome X [54].

It is believed that increased gene-body methylation correlates with increased transcription [30, 31, 37]; although some researchers have proposed that intragenic methylation might reduce gene expression [42]. Our data suggested that methylation in the gene body reduces gene expression (Figure 4). However, gene-body methylation is only one of the many factors that influence gene expression. Further studies focusing on the methylation of specific regions that exhibit distinct gene regulation contexts are needed to elucidate the complicated epigenetic mechanism underlying aging and its associations with disease.

Previous reports have indicated that increased protein catabolism occurs in aging skeletal muscles [55, 56]. Structural and functional changes associated with aging, such as reductions in the muscle mass and muscle fibers, have been observed across a wide range of species, from worms to mammals [14]. This type of epigenetic alteration of skeletal muscle with aging (Figure 6A and 6B; Additional file 10), consistent with findings in other species, was observed in pigs for the first time in the present work. Interestingly, genes related to tumorigenesis, as well as insulin sensitivity, exhibited a relatively higher expression level in the skeletal muscle of the MA pigs compared with younger pigs (Figure 6C), suggesting a higher risk of developing diseases with increased age.

Our results will promote further development of the pig as a model organism for human aging research. Most of the studies carried out in pigs to date have been conducted in neonatal or very young animals (generally when the pigs reach peak commercial value at approximately 6 months of age), before they reach the age of 1 year [57, 58]. Limited studies have been carried out on relatively older pigs (aged 2 years or more). Here, younger (0.5 years old) and middle-aged (7 years old) pigs were examined to investigate DNA methylation changes during the aging process. Although the aging process differs across species, with human aging showing major differences from the aging of most other species because of the relatively longer lifespan of humans, many species, including humans, pigs and mice, exhibit similarities with respect to aging muscle, muscular protein catabolism and muscle atrophy [59]. However, the time course of the muscle function changes occurring in pigs remains to be determined in further studies. In addition, pigs have a longer lifespan than that of rodents and present similar metabolic features, cardiovascular systems and proportional organ sizes to those humans [7]. Consequently, pigs can serve as a good biomedical model for human studies on the chronic aging process and its associated diseases [6, 8, 9]. However, only two age groups: young and middle-aged pigs were used in our study, and examining pigs of additional consecutive ages is necessary to further elucidate the changes in epigenetic modifications associated with age, as well as the ultimately complicated mechanisms underlying the aging process.

## Conclusions

In summary, the present study provides a comprehensive analysis of genome-wide DNA methylation patterns in the skeletal muscle of aging pigs. We identified remarkable DNA methylation changes, such as a tendency toward hypomethylation in gene bodies in the *longissimus dorsi* muscle of MA pigs. Furthermore, we identified numerous genes that exhibited age-related methylation changes and are potentially involved in the aging process. These genes are mainly related to protein catabolism, suggesting that predisposition to amyotrophy emerges during middle age. This study will serve as a valuable resource for aging studies, promoting the pig as a model organism for human aging research and accelerating the development of comparative animal models in aging research.

## Methods

### Animals

A total of six healthy female pigs (Chinese Jinhua breed) were used in this study from two age groups: 0.5 and 7 years old, representing young and middle-aged pigs, respectively. Each age group included three individuals, which were regarded as biological replicates. The animals were reared in the same environment and fed the same diet *ad libitum* during the experimental period. Food was withheld from the animals on the night before they were slaughtered. All experimental procedures and sample collection were approved by the Institutional Animal Care and Use Committee of the College of Animal Science and Technology of Sichuan Agricultural University, Sichuan, China, under permit No. DKY-B20121403.

### Tissue collection

Six types of tissues (brain, liver, ovary, spleen, heart and *longissimus dorsi* muscle) were rapidly sampled from each carcass and immediately frozen in liquid nitrogen. All tissue samples were stored at − 80°C until DNA and total RNA extraction.

### Measurement of the global DNA methylation status

DNA from each collected tissue was extracted using the DNeasy Blood & Tissue Kit (Qiagen). Global DNA methylation was evaluated using the MethylFlash™ Methylated DNA Quantification Kit (Epigentek). The amount of input DNA for each assay was 100 ng to ensure optimal quantification. The experiments were performed according to the manufacturer’s instructions.

### Quantitative PCR analysis of the *DNMT*genes

Total RNA (10 μg) was extracted from the six muscle samples using TRIzol (Invitrogen). RNase-free DNase I (TaKaRa) was used to remove genomic DNA from the RNA samples. cDNA was synthesized using PrimeScript RT Master Mix (TaKaRa). Quantitative real-time PCR (q-PCR) was performed using SYBR Premix Ex Taq (TaKaRa) in the CFX96 Real-Time PCR Detection System (Bio-Rad). The primers used for q-PCR are listed in Additional file 11. All measurements were performed in parallel with a negative control (no cDNA template), and each RNA sample was analyzed in triplicate. Porcine *ACTB*, *TBP* and *TOP2B* were used as endogenous control genes [60, 61]. The gradient dilution PCR assays for these three reference genes showed stable, high amplification efficiencies (Additional file 12). Relative expression levels were then calculated using the ΔΔCt method [62].

### Methylated DNA immunoprecipitation sequencing

DNA was extracted using the DNeasy Blood & Tissue Kit (Qiagen) and then eluted using 10 mM Tris•Cl, pH 7.5. The quality of the isolated DNA was then measured using a NanoDrop spectrophotometer. The ratio of the absorbance at 260 nm versus 280 nm (A260/A280) provides an estimate of the purity of the DNA. The A260/A280 value should be 1.8 to 2.0 for each DNA sample to guarantee quality. The initial volume of DNA for each sample should be at least 5 μg to ensure the success of subsequent MeDIP-seq experiments. The protocol for MeDIP-seq and detailed information on the construction of a MeDIP DNA library were provided in a previous report from our group [8]. The MeDIP-seq data have been submitted to the GEO database under accession number GSE50716.

### Analysis of MeDIP-seq data

We filtered out reads that contained more than 5 ‘N’s and those in which over 50% of the sequence exhibited a low quality value (Phred score < 5). The sequencing reads were then aligned to the pig reference genome version 9.2 with up to four mismatches allowed, using SOAP2 software (Version 2.21) [63]. We employed the Nov. 2009 (SGSC Sscrofa9.2/susScr2) assembly of the pig genome [64] (susScr2, SGSC Sscrofa9.2 (NCBI project 10718, GCA_000003025.2)), and the reference can be downloaded at http://hgdownload.cse.ucsc.edu/goldenPath/susScr2/chromosomes/. When multiple reads from one sequencing library were mapped to the same genomic location, these reads were regarded as potential clonal duplicates arising from PCR amplification biases and were identified to be a single read. The coverage depth for each CpG site was calculated from the number of DNA fragments covering that site, which were paired-end mapped reads, and further normalization among samples was performed based on the total DNA fragments for each sample. To avoid stochastic sampling drift and increase the confidence of the results, we filtered out CpG sites whose coverage showed a read depth of less than 10 when performing subsequent differential methylation analyses.

We also classified all of the genomic regions into 24 genomic elements while referring to annotation data for the pig reference genome to perform further detailed analyses according to previous studies [8]. It should be noted that the TSS for many porcine genes are incomplete because of genome assembly and annotation issues related to the pig genome. We arbitrarily defined the region extending from − 2,200 to + 500 bp from the gene translation start site as the promoter. All promoters (−2,200 to + 500 bp) were classified into three groups based on their CpG content: high CpG promoters (HCPs) that contained a 500-bp area with a CpG ratio above 0.75 and a GC content above 55%; low CpG promoters (LCPs), with did not contain a 500-bp area with a CpG ratio above 0.48; and intermediate CpG promoters (ICPs), which were neither HCPs nor LCPs. Each promoter of 2,700 bp in length was then divided into three groups: distal (D), −1,000 to −2,200 bp; intermediate (I), −200 to −1,000 bp; and proximal (P), −200 to +500 bp. Consistent improvement of the assembly and annotation of the pig genome will further the search for epigenetic biomarkers of aging and promote development of the pig as a model organism for human biomedical research.

We classified CGIs (regions of at least 200 bp with a GC percentage greater than 55% and an observed-to-expected CpG ratio greater than 65%) and CGI shores (regions located within 2 kb of islands) into four classes: promoter, intragenic, 3’ transcript or intergenic locations, based on their distance from genes.

We also divided the gene structure into the first exon, first intron, internal exons, internal introns, and last exon, together with the TES (Transcription end site) 2 kb downstream and the intergenic region according to the pig genome (*Sus scrofa* 9.2).

### Identification of DMRs

After filtering out low-quality reads (reads with a depth of less than 10), we identified DMRs across the whole genome using methods we have described previously [8] to identify the differential DNA methylation status between the two age groups. First, the normality and equal variance of the read depth at each CpG site across the sample groups were tested using Bartlett's test (passing if *P* > 0.05, failing if *P* < 0.05). Second, a parametric (when passing Bartlett's test) or non-parametric test (when failing Bartlett's test) was used to select highly variable CpGs (*P* < 0.01) as seed sites for candidate DMRs. Third, the 3’ downstream adjacent CpG sites were individually incorporated into the seed CpGs. To highlight the CpG-enriched regions, we allowed a distance of up to 200 bp between two adjacent CpGs. The average read depth of these two CpGs was then subjected to a new round of tests, which was continued repeatedly for the next CpG until a low-variance CpG (*P* > 0.01) was encountered, which was allowed to be up to 2 kb from the seed CpG. If five or more CpGs in a genomic region showed different read depths across samples that were statistically significant (*P* < 0.01), then the region was considered a DMR. The resulting *P* values for the DMRs were corrected using the Benjamini-Hochberg method (FDR < 0.01, 1,000 permutations).

### Measurement of telomere length using q-PCR

The high-quality DNA used in the MeDIP experiment was also used to measure telomere length in the young and MA pigs. The average telomere length was measured from a real-time PCR assay, following a previous description [36, 65]. Two separate PCR assays were performed, using a telomeric region primer (T) and a primer for a reference nuclear gene (pig *GCG*, single copy gene, S). The primer sequences were as follows: T forward, CGGTTTGTTTGGGTTTGGGTTTGGGTTTGGGTTTGGGTT; T reverse, GGCTTGCCTTACCCTTACCCTTACCCTTACCCTTACCCT; S forward, GAATCAACACCATCGGTCAAAT; and S reverse, CTCCACCCATAGAATGCCCAGT. The telomere (T) signal was normalized to the signal of the single-copy (S) gene to generate the T/S ratio, which reflected the relative telomere length, in all studied samples.

### Functional enrichment analysis for genes with DMRs

The DAVID (Database for Annotation, Visualization and Integrated Discovery) web server (http://david.abcc.ncifcrf.gov/) was used to perform functional enrichment analysis of Gene Ontology (GO) and KEGG pathway categories [66]. Genes with DMRs in their promoters and gene bodies were mapped to their respective human orthologs and then submitted to DAVID for enrichment analysis, which included GO biological processes (GO-BP), molecular function (GO-MF) terminologies and KEGG pathway categories. Only GO-BP, GO-MF or KEGG-pathway terms with a Benjamini-corrected *P* value less than 0.05 were considered to be significant and therefore included in the list.

### Gene expression microarray

Total RNA (10 μg) was extracted from the six samples using TRIzol (Invitrogen) and further purified using an RNeasy column (Qiagen). The integrity of the total RNA was confirmed using a Bioanalyzer 2100 and the RNA 6000 Nano LabChip Kit (Agilent Technologies). Detailed information on the workflow of the microarray experiment is provided in a previous report from our group [8]. First, we mapped 43,603 probes (60 mer in length) to the pig reference genome while allowing up to one mismatch, which resulted in 27,955 probes (64.11%) that were uniquely mapped. Among these uniquely mapped probes, 4,983 (11.43%) were uniquely mapped to exons in Ensembl genes (more than 60% sequence overlap). Multiple probes that mapped to the same or different exons of a specific gene were excluded. Therefore, only 3,074 probes (7.05%), which uniquely represented 3,074 genes and were considered to represent high-confidence gene expression data, were used in the subsequent analysis.

Differentially expressed genes were identified using the MultiExperiment Viewer (MeV) [67], and this software was also employed to perform subsequent hierarchical clustering of samples. The gene expression microarray data have been submitted to the GEO database under accession number GSE49791.

### Bisulfite sequencing PCR

Methylation Primer Express Software V1.0 was used to design bisulfite sequencing PCR (BSP) primers, which are provided in Additional file 13. The bisulfite conversion of genomic DNA was performed using the EZ DNA Methylation-Gold™ Kit (Zymo Research, D5006). PCR was carried out using ZymoTaq™ PreMix (Zymo Research, E2004). The PCR product was then purified using the DNA Clean & Concentrator - 25™ Kit (Zymo Research, D4005), and the PCR product was cloned into the TA vector pCR2.1 (Invitrogen, K2000-01). Ten subclones were selected for each gene and subsequently sequenced using an ABI 3730 DNA sequencer (Applied Biosystems). All of the sequences were analyzed using BiQ Analyzer V2.0 software [68].

## Electronic supplementary material

Additional file 1: **Relative mRNA expression levels of** ***DNMTs*** **in six tissues between young and middle-age pigs.** The expression levels were normalized to the maximum value obtained in the two groups. (Student’s *t*-test, **P < 0.01, *P < 0.05). (PDF 479 KB)

Additional file 2: **Summary of MeDIP-seq data production.** Low-quality reads were filtered out of the raw reads, and the clean reads were then used in further analyses. “% aligned” is the percentage of clean reads aligned to the pig reference genome (version 9.2). “% unique” is the percentage of reads uniquely aligned across all of the aligned reads. The reads showing the same mapping locations in each sample were considered to be potentially duplicated clones generated via PCR amplification during sequencing library construction and were therefore removed from the analysis. “% non-duplicate alignment” is the percentage of uniquely aligned non-duplicated reads over all of the uniquely aligned reads. (PDF 394 KB)

Additional file 3: **Percentage of CpGs showing an average coverage that meets the read depth threshold over all samples.** Values are the means ± s.d. (*n* = 6). (PDF 360 KB)

Additional file 4: **Genome-wide distribution of the DNA methylation levels.** To compare the DNA methylation state between samples, the read depth was normalized to the overall average number of reads in each group. The CpG_o/e_ ratio, SNPs density, numbers of genes, repeats and CGIs were all calculated over 1 M Mb sliding windows. (PDF 2 MB)

Additional file 5: **Pearson’s correlation between DNA methylation levels and chromosomal features.** (PDF 403 KB)

Additional file 6: **Box plots of the percentages of GC content in the promoter, gene-body, and intergenic regions.** Box-plot edges indicate the 25^th^ and 75^th^ percentiles; central bars indicate the medians; and whiskers indicate the non-outlier extremes. The significance was evaluated using *t*-tests. (PDF 205 KB)

Additional file 7: **Comparison of methylation levels in various categories of genomic elements.** The average methylation level for each group was plotted. Then DNA methylation levels (average read depth of two age groups) of various categories were compared. (A) Methylation levels across various promoter categories were compared using two-way ANOVA. Intermediate CpG promoters (ICPs) exhibited a relatively higher methylation status relative to those of high CpG promoters (HCPs) and low CpG promoters (LCPs) (*P =* 2.87 × 10^−37^). The methylation levels within the distal (D), proximal (P) and intermediate (I) regions also showed significant differences (*P* =1.44 × 10^−7^). (B) Comparison of methylation levels between exons and introns was performed using Student’s *t*-test. The exon methylation was higher than the intron methylation (*P =* 9.8 × 10^−9^). (C) Methylation levels between CGIs and CGI shores were compared using two-way ANOVA. The average methylation in CGI shores was significantly higher than that in CGIs (*P =* 2.17 × 10^−3^). (PDF 595 KB)

Additional file 8: **Hierarchical clustering of samples using DMRs in various genomic elements and differentially expressed (DE) mRNAs.** Clustering was performed using MultiExperiment Viewer software. The distance metric applied for clustering was Pearson correlation across samples. (PDF 341 KB)

Additional file 9: **List of DMRs.** (XLS 2 MB)

Additional file 10: **Over-represented functional gene categories for DMRs.** (XLS 49 KB)

Additional file 11: **Information on primers used to perform q-PCR.** (PDF 396 KB)

Additional file 12: **Amplification efficiencies of gradient dilution PCR assays for the** ***ACTB,*** ***TBP*** **and** ***TOP2B*** **genes.** (PDF 393 KB)

Additional file 13: **Information on primers used to perform BSP.** (PDF 342 KB)

## Abbreviations

LDM: *Longissimus dorsi* muscle
MA: Middle-aged
DMRs: Differentially methylated regions
DNMTs: DNA methyltransferases
MeDIP-seq: Methylated DNA immunoprecipitation sequencing
HCP: High CpG promoter
ICP: Intermediate CpG promoter
LCP: Low CpG promoter
CGI: CpG island
D: Distal
P: Proximal
I: Intermediate
HAGR: Human ageing genomic resources
BSP: Bisulfite sequencing PCR.
