# Supplementary material for: Genome-wide DNA methylation changes in skeletal muscle between young and middle-aged pigs
Source: BMC Genomics. 2014 Aug 5;15(1):653. doi: 10.1186/1471-2164-15-653 (PMC4147169; doi:10.1186/1471-2164-15-653)
Supplement: Supplementary file 1 — Additional file 1: Relative mRNA expression levels of DNMTs in six tissues between young and middle-age pigs. The expression levels were normalized to the maximum value obtained in the two groups. (Student’s t-test, **P < 0.01, *P < 0.05). (PDF 479 KB) [file 12864_2014_6371_MOESM1_ESM.pdf]

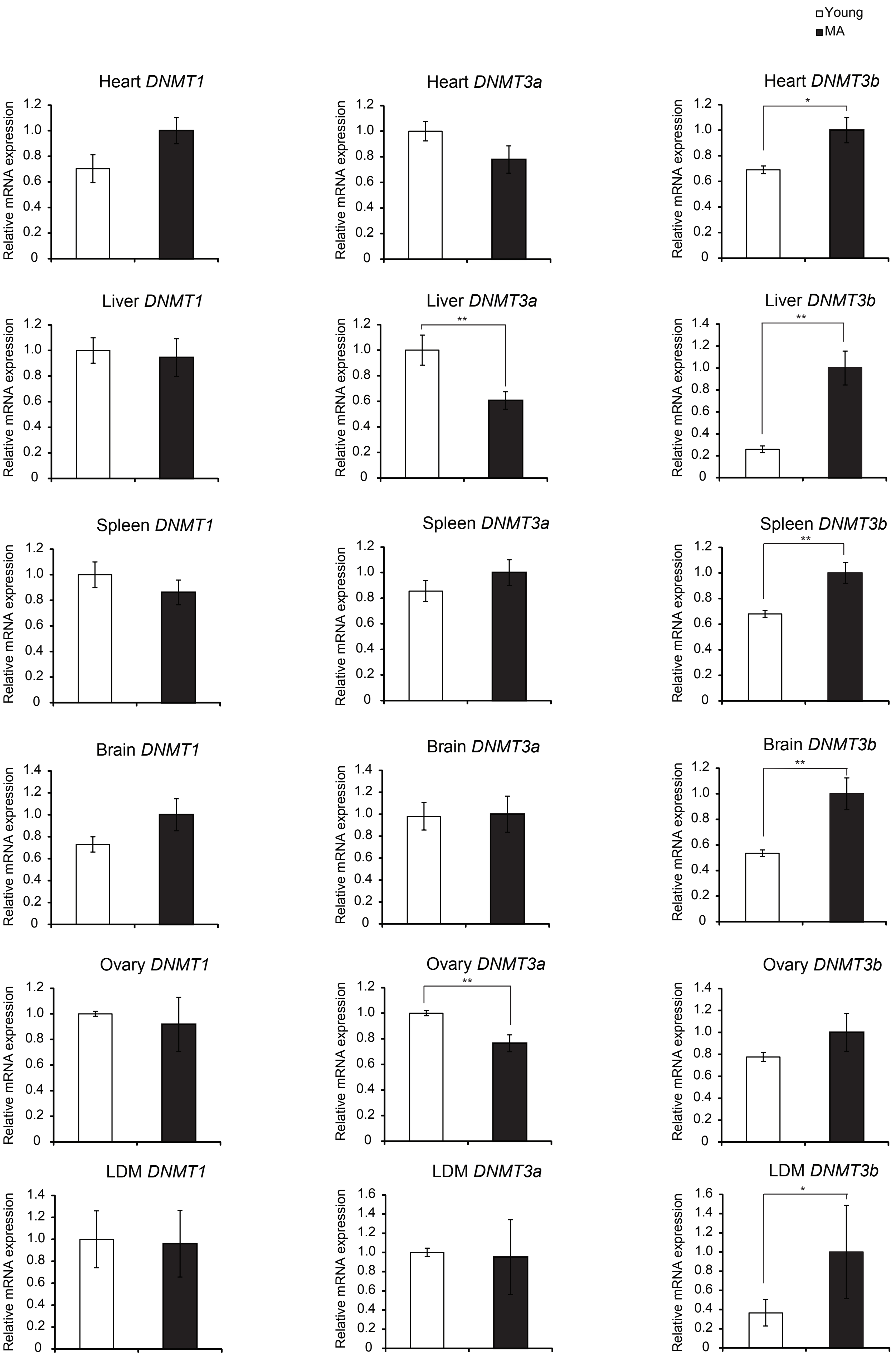

Additional file 1: Relative mRNA expression levels of *DNMTs* in six tissues between young and middle-age pigs. The expression levels were normalized to the maximum value obtained in the two groups. (Student's *t*-test, $^{**}P < 0.01$, $^{*}P < 0.05$)
