# Supplementary material for: Genome-wide DNA methylation changes in skeletal muscle between young and middle-aged pigs
Source: BMC Genomics. 2014 Aug 5;15(1):653. doi: 10.1186/1471-2164-15-653 (PMC4147169; doi:10.1186/1471-2164-15-653)
Supplement: Supplementary file 2 — Additional file 2: Summary of MeDIP-seq data production. Low-quality reads were filtered out of the raw reads, and the clean reads were then used in further analyses. “% aligned” is the percentage of clean reads aligned to the pig reference genome (version 9.2). “% unique” is the percentage of reads uniquely aligned across all of the aligned reads. The reads showing the same mapping locations in each sample were considered to be potentially duplicated clones generated via PCR amplification during sequencing library construction and were therefore removed from the analysis. “% non-duplicate alignment” is the percentage of uniquely aligned non-duplicated reads over all of the uniquely aligned reads. (PDF 394 KB) [file 12864_2014_6371_MOESM2_ESM.pdf]

Additional file 2: Summary of MeDIP-seq data production.

Tissue symbol	Biological replicate	Number of raw reads	Raw reads (Gb)	Number of clean reads	Clean reads (Gb)	% aligned	% unique	% non-duplicate alignment
**Young**	**1**	138,296,328	6.78	131,316,922	6.43	79.41	74.39	94.85
	**2**	151,439,856	7.42	145,699,912	7.14	80.54	74.97	93.82
	**3**	144,787,884	7.09	140,672,846	6.89	82.57	77.36	90.64
**MA**	**1**	170,671,802	8.36	161,873,526	7.93	80.77	74.64	92.22
	**2**	178,619,948	8.75	168,877,592	8.28	80.84	74.4	91.53
	**3**	152,207,228	7.46	144,007,480	7.06	80.33	75.65	89.86
	**Total**	**936,023,046**	**46**	**892,448,278**	**44**	**81**	**75**	**92**
