# Supplementary material for: Genome-wide DNA methylation changes in skeletal muscle between young and middle-aged pigs
Source: BMC Genomics. 2014 Aug 5;15(1):653. doi: 10.1186/1471-2164-15-653 (PMC4147169; doi:10.1186/1471-2164-15-653)
Supplement: Supplementary file 3 — Additional file 3: Percentage of CpGs showing an average coverage that meets the read depth threshold over all samples. Values are the means ± s.d. (n = 6). (PDF 360 KB) [file 12864_2014_6371_MOESM3_ESM.pdf]

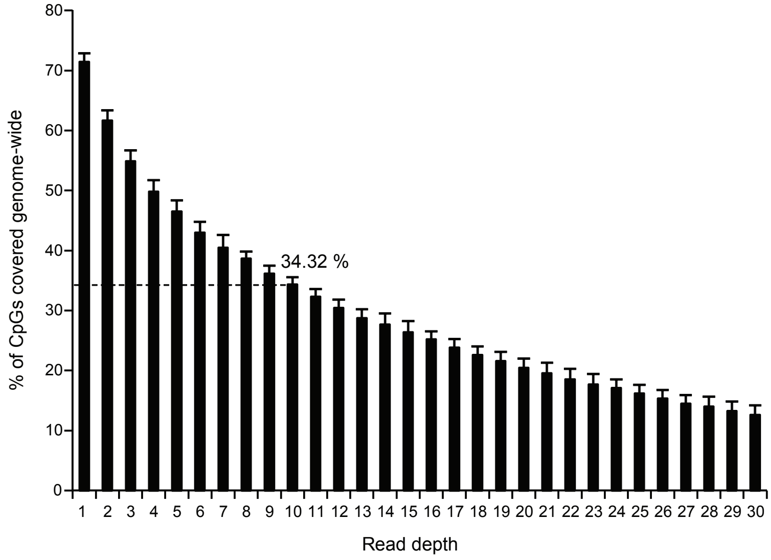

Additional file 3: Percentage of CpGs showing an average coverage that meets the read depth threshold over all samples. Values are the means $\pm$ s.d. (n = 6).
