# Supplementary material for: Genome-wide DNA methylation changes in skeletal muscle between young and middle-aged pigs
Source: BMC Genomics. 2014 Aug 5;15(1):653. doi: 10.1186/1471-2164-15-653 (PMC4147169; doi:10.1186/1471-2164-15-653)
Supplement: Supplementary file 4 — Additional file 4: Genome-wide distribution of the DNA methylation levels. To compare the DNA methylation state between samples, the read depth was normalized to the overall average number of reads in each group. The CpGo/e ratio, SNPs density, numbers of genes, repeats and CGIs were all calculated over 1 M Mb sliding windows. (PDF 2 MB) [file 12864_2014_6371_MOESM4_ESM.pdf]

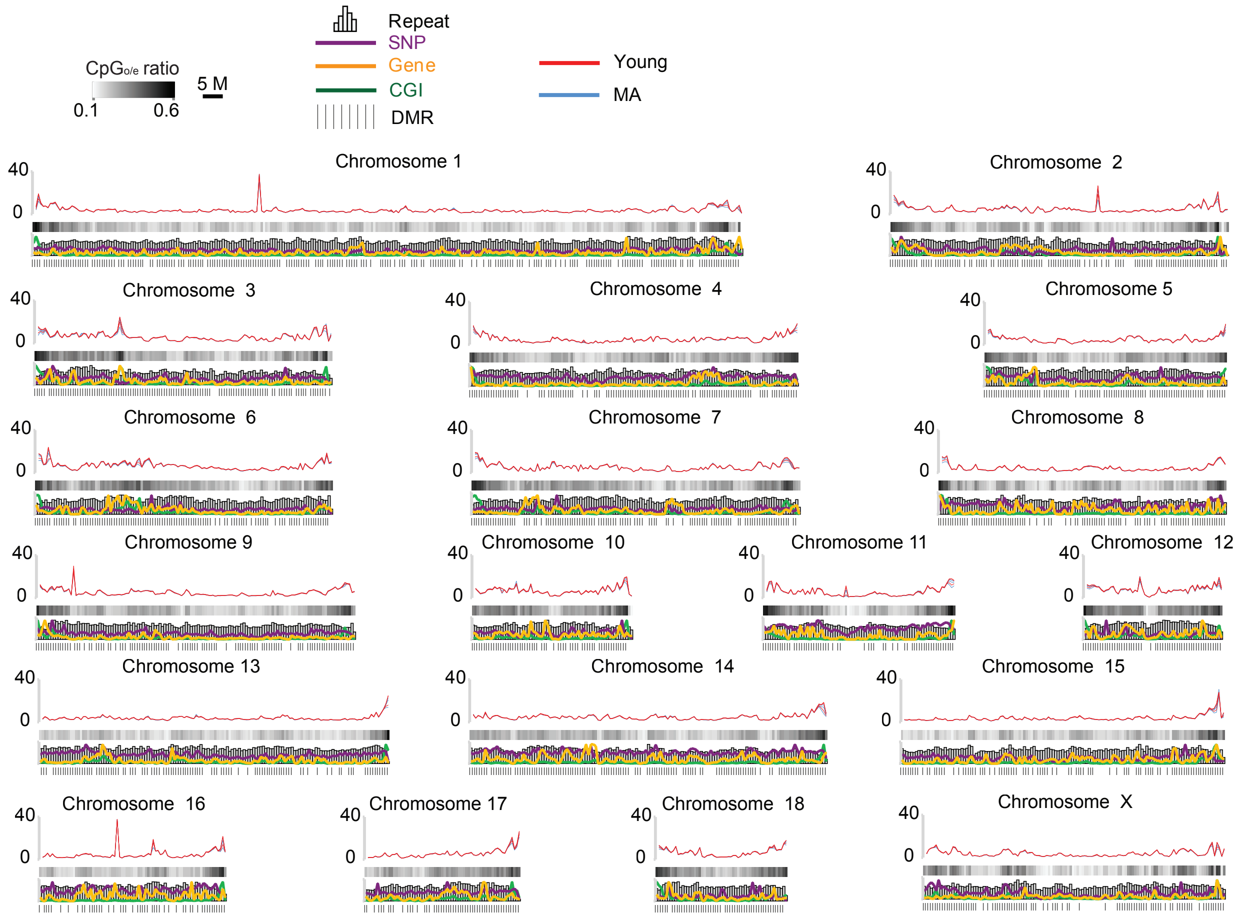

Additional file 4: Genome-wide distribution of the DNA methylation levels. To compare the DNA methylation state between samples, the read depth was normalized to the overall average number of reads in each group. The $CpG_{o/e}$ ratio, SNPs density, numbers of genes, repeats and CGIs were all calculated over 1 M Mb sliding windows.
