# Supplementary material for: Genome-wide DNA methylation changes in skeletal muscle between young and middle-aged pigs
Source: BMC Genomics. 2014 Aug 5;15(1):653. doi: 10.1186/1471-2164-15-653 (PMC4147169; doi:10.1186/1471-2164-15-653)
Supplement: Supplementary file 5 — Additional file 5: Pearson’s correlation between DNA methylation levels and chromosomal features. (PDF 403 KB) [file 12864_2014_6371_MOESM5_ESM.pdf]

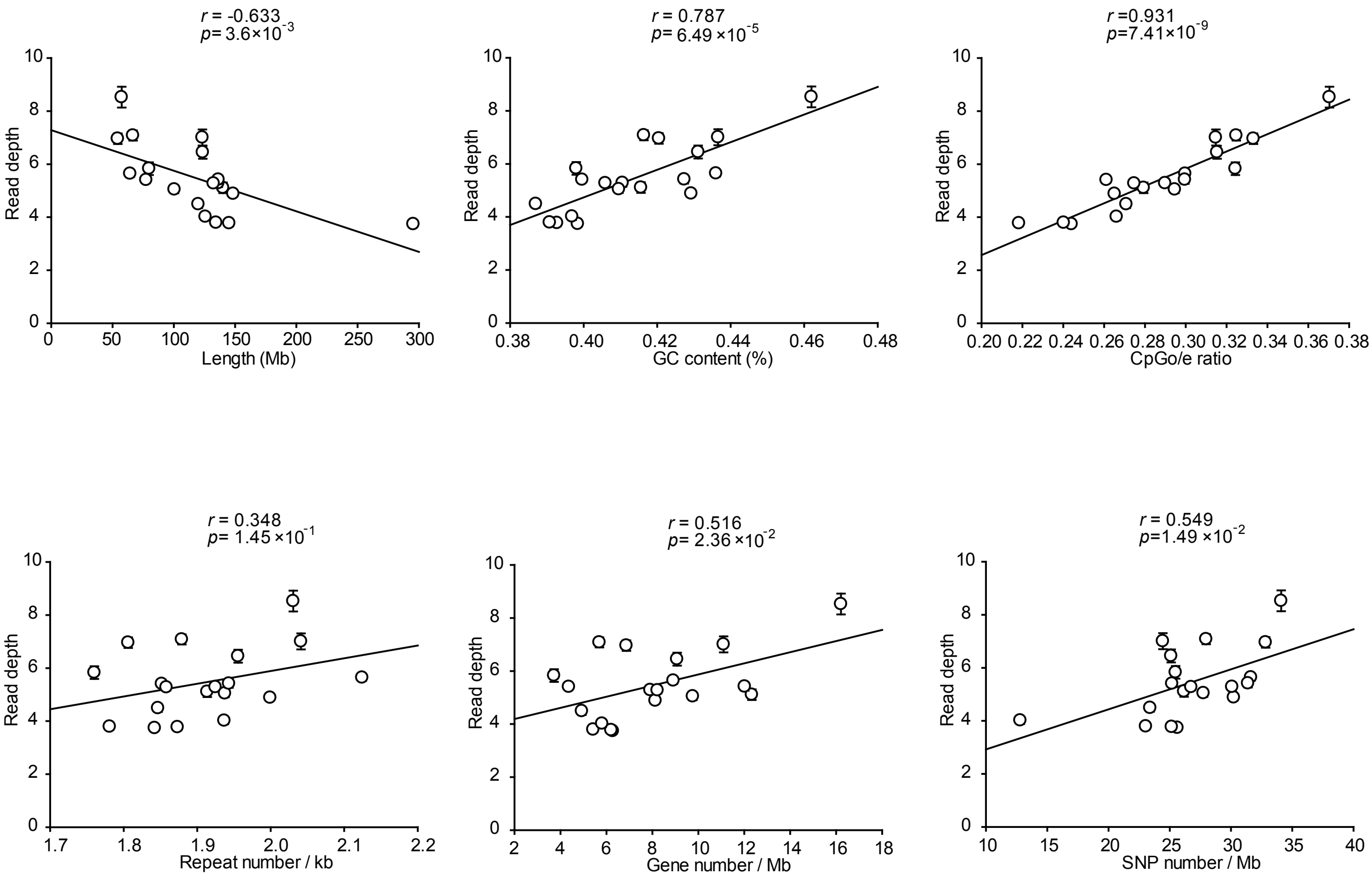

Additional file 5: Pearson's correlation between DNA methylation level and chromosomal features.
