# Supplementary material for: Genome-wide DNA methylation changes in skeletal muscle between young and middle-aged pigs
Source: BMC Genomics. 2014 Aug 5;15(1):653. doi: 10.1186/1471-2164-15-653 (PMC4147169; doi:10.1186/1471-2164-15-653)
Supplement: Supplementary file 6 — Additional file 6: Box plots of the percentages of GC content in the promoter, gene-body, and intergenic regions. Box-plot edges indicate the 25th and 75th percentiles; central bars indicate the medians; and whiskers indicate the non-outlier extremes. The significance was evaluated using t-tests. (PDF 205 KB) [file 12864_2014_6371_MOESM6_ESM.pdf]

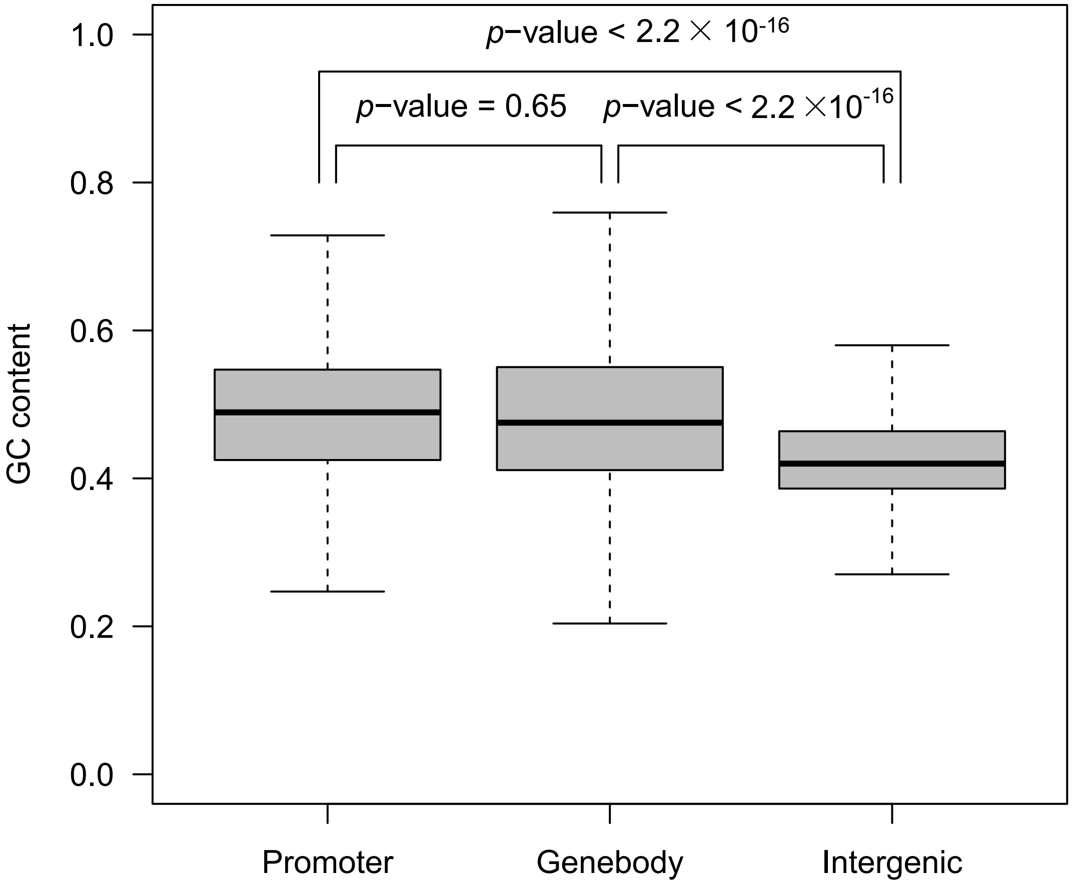

Additional file 6: Box plots of the percentages of GC content in the promoter, gene-body, and intergenic regions. Box-plot edges indicate the 25th and 75th percentiles; central bars indicate the medians; and whiskers indicate the non-outlier extremes. The significance was evaluated using *t*-tests.
