# Supplementary material for: Genome-wide DNA methylation changes in skeletal muscle between young and middle-aged pigs
Source: BMC Genomics. 2014 Aug 5;15(1):653. doi: 10.1186/1471-2164-15-653 (PMC4147169; doi:10.1186/1471-2164-15-653)
Supplement: Supplementary file 7 — Additional file 7: Comparison of methylation levels in various categories of genomic elements. The average methylation level for each group was plotted. Then DNA methylation levels (average read depth of two age groups) of various categories were compared. (A) Methylation levels across various promoter categories were compared using two-way ANOVA. Intermediate CpG promoters (ICPs) exhibited a relatively higher methylation status relative to those of high CpG promoters (HCPs) and low CpG promoters (LCPs) (P = 2.87 × 10^−37^). The methylation levels within the distal (D), proximal (P) and intermediate (I) regions also showed significant differences (P =1.44 × 10^−7^). (B) Comparison of methylation levels between exons and introns was performed using Student’s t-test. The exon methylation was higher than the intron methylation (P = 9.8 × 10^−9^). (C) Methylation levels between CGIs and CGI shores were compared using two-way ANOVA. The average methylation in CGI shores was significantly higher than that in CGIs (P = 2.17 × 10^−3^). (PDF 595 KB) [file 12864_2014_6371_MOESM7_ESM.pdf]

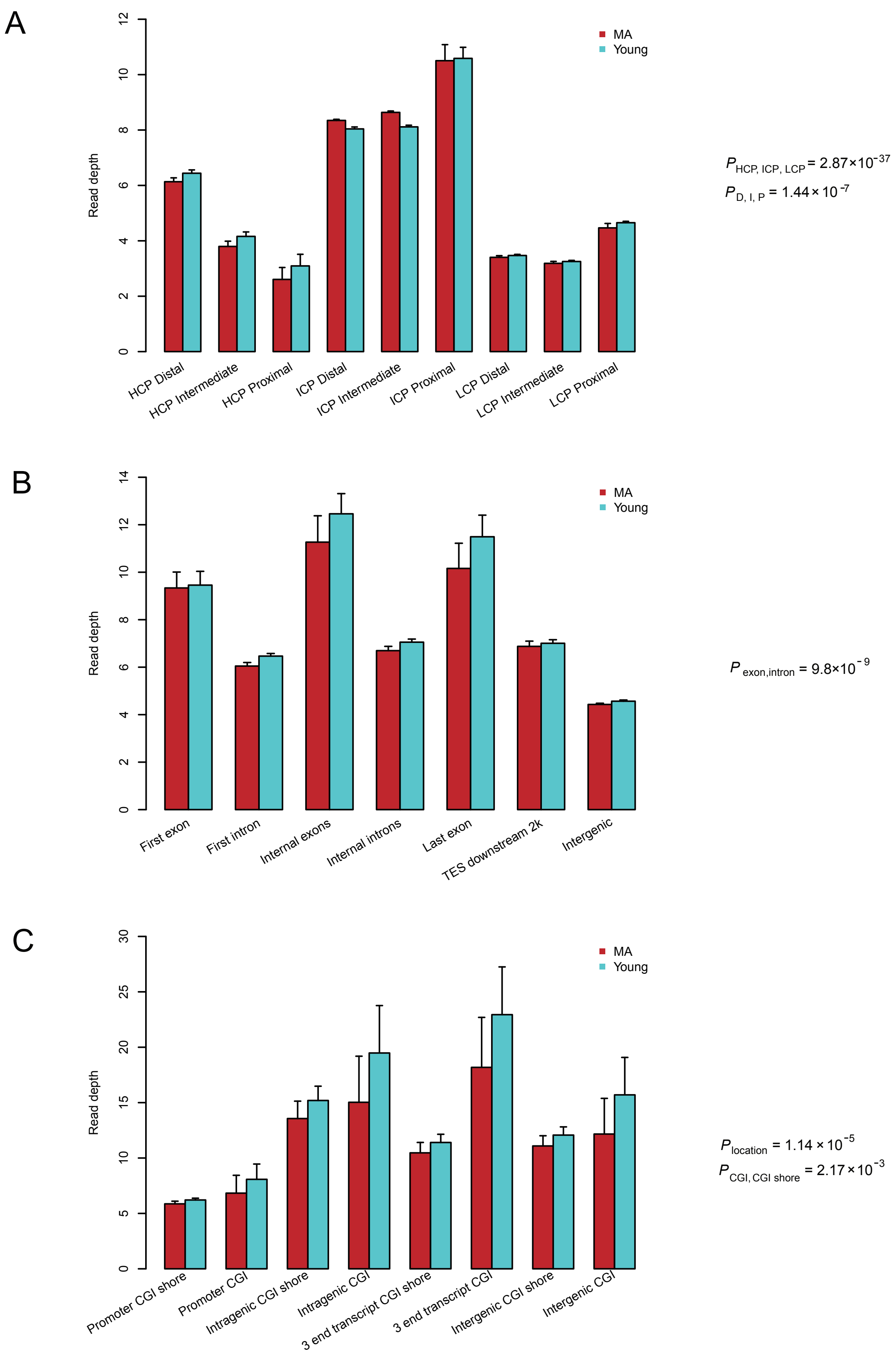

Additional file 7: Comparison of methylation levels in various categories of genomic elements. The methylation level for each sample was plotted. The average methylation level for each group was plotted. Then DNA methylation levels (average read depth of two age groups) of various categories were compared. (A) Methylation levels across various promoter categories were compared using two-way ANOVA. Intermediate CpG promoters (ICPs) exhibited a relatively higher methylation status relative to those of high CpG promoters (HCPs) and low CpG promoters (LCPs) ($P = 2.87 \times 10^{-37}$). The methylation levels within the distal (D), proximal (P) and intermediate (I) regions also showed significant differences ($P = 1.44 \times 10^{-7}$). (B) Comparison of methylation levels between exons and introns was performed using Student's *t*-test. The exon methylation was higher than the intron methylation ($P = 9.8 \times 10^{-9}$). (C) Methylation levels between CGIs and CGI shores were compared using two-way ANOVA. The average methylation in CGI shores was significantly higher than that in CGIs ($P = 2.17 \times 10^{-3}$).
