# Supplementary material for: Genome-wide DNA methylation changes in skeletal muscle between young and middle-aged pigs
Source: BMC Genomics. 2014 Aug 5;15(1):653. doi: 10.1186/1471-2164-15-653 (PMC4147169; doi:10.1186/1471-2164-15-653)
Supplement: Supplementary file 8 — Additional file 8: Hierarchical clustering of samples using DMRs in various genomic elements and differentially expressed (DE) mRNAs. Clustering was performed using MultiExperiment Viewer software. The distance metric applied for clustering was Pearson correlation across samples. (PDF 341 KB) [file 12864_2014_6371_MOESM8_ESM.pdf]

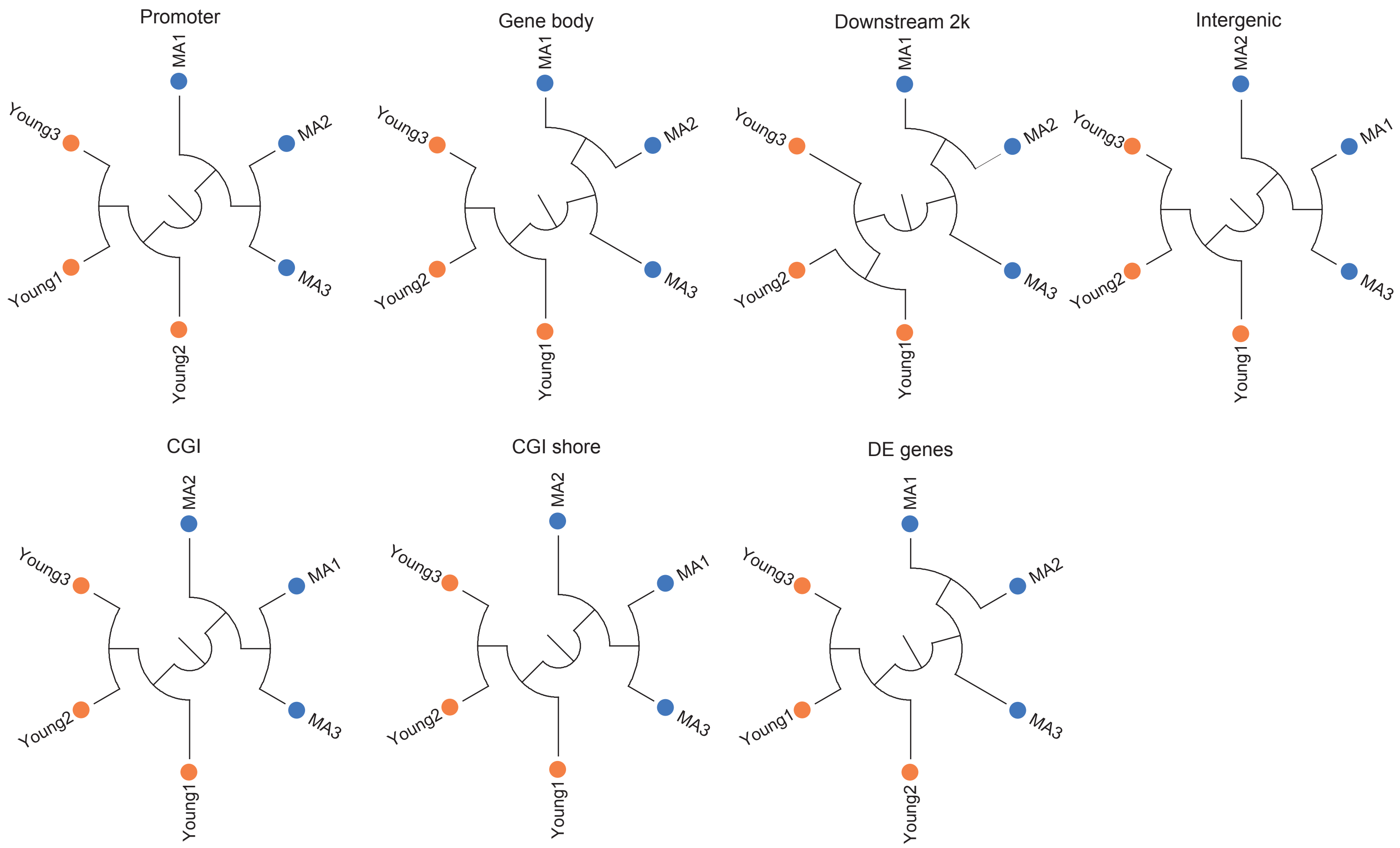

Additional file 8: Hierarchical clustering of samples using DMRs in various genomic elements and differentially expressed (DE) mRNAs. Clustering was performed using MultiExperiment Viewer software. The distance metric applied for clustering was Pearson correlation across samples.
