# Supplementary material for: Genome-wide DNA methylation changes in skeletal muscle between young and middle-aged pigs
Source: BMC Genomics. 2014 Aug 5;15(1):653. doi: 10.1186/1471-2164-15-653 (PMC4147169; doi:10.1186/1471-2164-15-653)
Supplement: Supplementary file 11 — Additional file 11: Information on primers used to perform q-PCR. (PDF 396 KB) [file 12864_2014_6371_MOESM11_ESM.pdf]

Additional file 11: Information on primers used to perform q-PCR.

Gene symbol	Primer sequence(5′ →3′)	Amplicon length/bp	Annealing temperature (°C)	GenBank No.
*ACTB**	TCTGGCACCACACCTTCT<br>TGATCTGGGTCATCTTCTCAC	114	60.0	DQ178122
*TBP**	GATGGACGTTCGGTTTAGG<br>AGCAGCACAGTACGAGCAA	124	60.0	DQ178129
*TOP2B**	AACTGGATGATGCTAATGATGCT<br>TGGAAAAACTCCGTATCTGTCTC	137	60.0	AF222921
DNMT1	TGGCGGGACCTACCAAACA<br>ACTTCCACGCAGGAGCAGA	137	64.5	NM_001032355.1
DNMT3a	AAGAATGCCACCAAATCAGCC<br>AGAACTTGCCGTCTCCGAACCA	196	63.3	NM_001097437.1
DNMT3b	AGGTCTCCAGCCTCCTAAGTT<br>GTGTCTGAGCCATCTCCATCC	82	57.0	DQ830979.1

* denotes the endogenous control genes
