# Supplementary material for: Genome-wide DNA methylation changes in skeletal muscle between young and middle-aged pigs
Source: BMC Genomics. 2014 Aug 5;15(1):653. doi: 10.1186/1471-2164-15-653 (PMC4147169; doi:10.1186/1471-2164-15-653)
Supplement: Supplementary file 12 — Additional file 12: Amplification efficiencies of gradient dilution PCR assays for the ACTB, TBP and TOP2B genes. (PDF 393 KB) [file 12864_2014_6371_MOESM12_ESM.pdf]

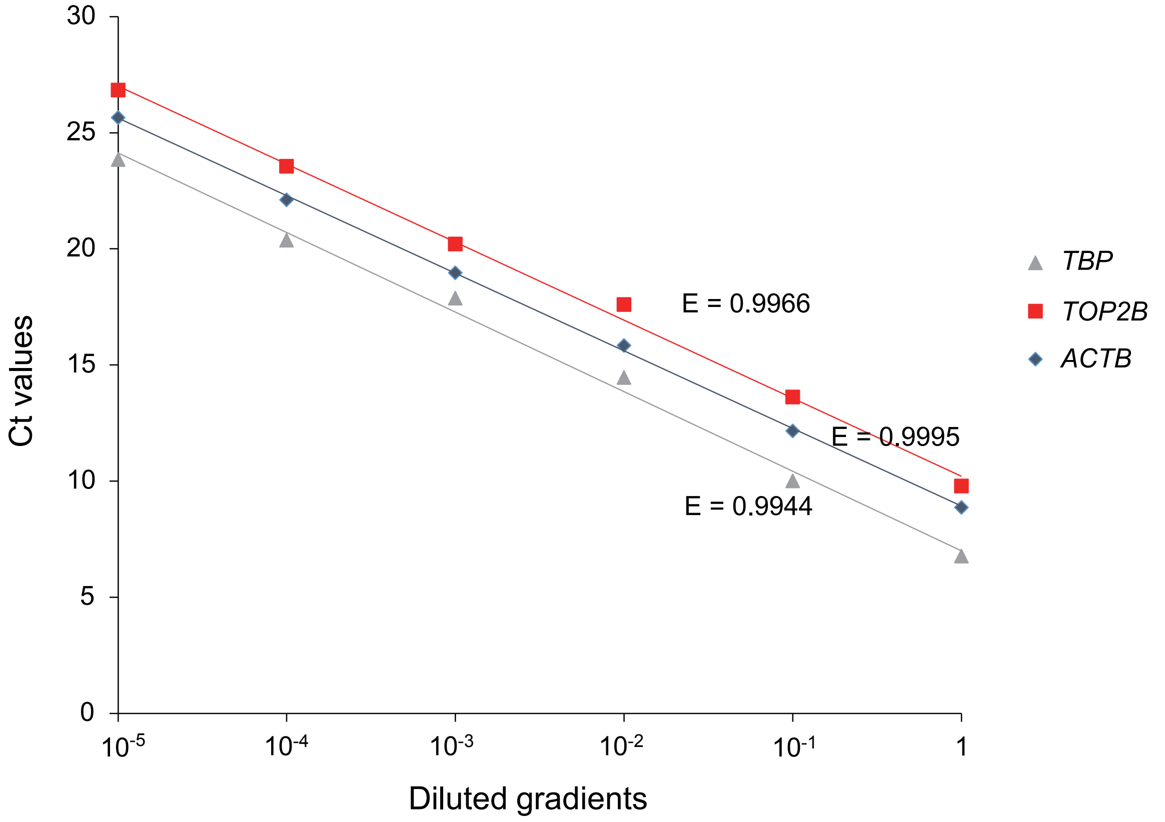

Additional file 12: Amplification efficiencies of gradient dilution PCR assays for the *ACTB*, *TBP* and *TOP2B* genes.
