# Supplementary material for: Genome-wide DNA methylation changes in skeletal muscle between young and middle-aged pigs
Source: BMC Genomics. 2014 Aug 5;15(1):653. doi: 10.1186/1471-2164-15-653 (PMC4147169; doi:10.1186/1471-2164-15-653)
Supplement: Supplementary file 13 — Additional file 13: Information on primers used to perform BSP. (PDF 342 KB) [file 12864_2014_6371_MOESM13_ESM.pdf]

Additional file 13: Information on primers used to perform BSP.

Gene	Primer sequence	PCR product length (bp)
FGFR1	AGTTTTTATATTTTAATGGGTTTTT	265
	ACTCAAACTATCCTCAAAATAAAAA	
FOXO3	TATTAGAAGATAGTGTTAGAGTAAGGAAATAA	278
	TTAAAAATCCAACATTACTACAAAATAT	
GRB2	TATTTTTGTTTGGAGAAAGAAAT	481
	AACAAAAAATAAAATCAATCTTCA	
